# Isolation of neuronal chromatin from brain tissue

**DOI:** 10.1186/1471-2202-9-42

**Published:** 2008-04-28

**Authors:** Yan Jiang, Anouch Matevossian, Hsien-Sung Huang, Juerg Straubhaar, Schahram Akbarian

**Affiliations:** 1 Brudnick Neuropsychiatric Research Institute, Department of Psychiatry, University of Massachusetts Medical School, Worcester, MA, USA; 2 Graduate School of Biomedical Sciences, University of Massachusetts Medical School, Worcester, MA, USA; 3 Program in Molecular Medicine, University of Massachusetts Medical School, Worcester, MA, USA

## Abstract

**Background:**

DNA-protein interactions in mature brain are increasingly recognized as key regulators for behavioral plasticity and neuronal dysfunction in chronic neuropsychiatric disease. However, chromatin assays typically lack single cell resolution, and therefore little is known about chromatin regulation of differentiated neuronal nuclei that reside in brain parenchyma intermingled with various types of non-neuronal cells.

**Results:**

Here, we describe a protocol to selectively tag neuronal nuclei from adult brain – either by (anti-NeuN) immunolabeling or transgene-derived histone H2B-GFP fusion protein – for subsequent fluorescence-activated sorting and chromatin immunoprecipitation (ChIP). To illustrate an example, we compared histone H3 lysine 4 and 9 methylation marks at select gene promoters in neuronal, non-neuronal and unsorted chromatin from mouse forebrain and human cerebral cortex, and provide evidence for neuron-specific histone methylation signatures.

**Conclusion:**

With the modifications detailed in this protocol, the method can be used to collect nuclei from specific subtypes of neurons from any brain region for subsequent ChIP with native/un-fixed or crosslinked chromatin preparations. Starting with the harvest of brain tissue, ChIP-ready neuronal nuclei can be obtained within one day.

## Background

An increasing number of neurodevelopmental and neuropsychiatric disorders are thought to result from defective DNA:protein interactions specifically in neurons; furthermore, sustained changes in neuronal gene expression and behavior after exposure to certain drugs or stimuli are likely to involve chromatin remodeling, including DNA methylation and histone modification changes [1-5]. However, even the most sensitive chromatin immunoprecipitation assays and most other approaches used to study the regulation of DNA and histone modifications, transcription factor binding etc., lack single cell resolution and instead require the preparation of homogenates from at least 10^3 ^– 10^7 ^nuclei. Consequently, detailed chromatin analysis was until now not feasible for nuclei of terminally differentiated neurons that typically reside in brain parenchyma intermingled with various types of glia and other cells mostly in a 2:1 – 1:2 ratio, dependent on species and brain regions [6,7].

Recently, immunostaining in conjunction with fluorescence-activated cell sorting (FACS) was used successfully to selectively collect neuronal nuclei from human (postmortem) brain tissue for the purposes of retrospective birth dating [8] or assessment of age-related changes in DNA cytosine methylation [9]. However, these studies utilized the nuclear harvest for highly sensitive radiation and PCR assays, and it remained unclear whether the protocol could be modified for the purposes of chromatin immunoprecipitation and other techniques that require comparatively larger amount of input (for example, 10^5 ^10^7 ^nuclei). We provide a detailed protocol for selective sorting of neuronal nuclei from mouse and human brain in quantities sufficient for immunoprecipitation with different chromatin preparations (enzyme-based digestion and crosslinking/sonication), followed by microarray or PCR studies. In addition, we introduce a transgenic mouse line for neuron-specific expression of GFP (enhanced green fluorescent protein)-tagged histone H2B. Evidence is presented that even under baseline conditions, promoter-bound histone methylation in neuronal samples is significantly different when compared to unsorted, or non-neuronal nuclei from the same brain region. Therefore, the methods presented here will be important for the study of molecular mechanisms governing epigenetic control of neuronal gene expression and chromatin remodeling specifically in mature brain.

## Results

### H2B-GFP transgenic mice

The promoter of the α subunit of the Ca^2+^/calmodulin dependent protein kinase II gene (CAMKII) was used to drive H2B-EGFP expression; as expected, this transgene labeled most of the neuronal populations in the fore- and midbrain, including cortex, striatum, hippocampus, with the notable exception of the GABAergic interneurons in cerebral cortex and hippocampus (Fig. 3). In contrast, labeling in hindbrain, incl cerebellum, was less consistent (data not shown). The transgene-derived labeling of neuronal nuclei with H2B-EGFP was robust pre- and post-FACS (Fig. 2, panel a-d). To date, our oldest transgenic mice are 5 months of age and so far we did not observe any overt neurological phenotypes, even in animals expressing the fusion protein at comparatively high levels in CNS neurons. Likewise, no adverse effects were reported for transgenic mice expressing high levels of H2B-EGFP in a wide range of tissues, including brain [10].

**Figure 1:**
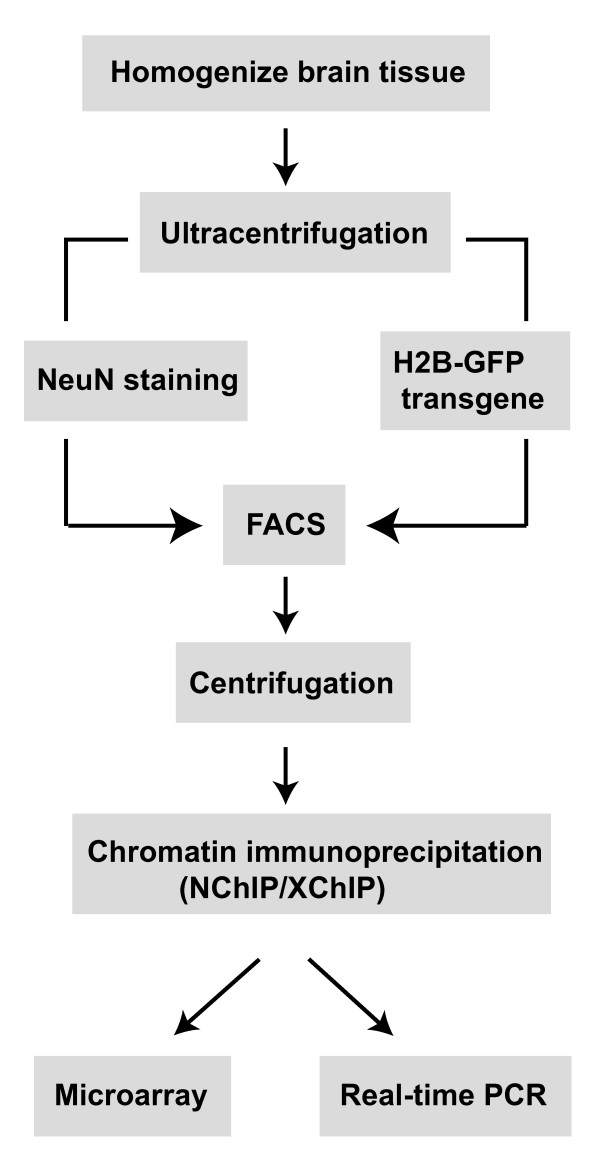
Outline of the procedure to isolate neuronal nuclei from brain for chromatin immunoprecipitation.

**Figure 2:**
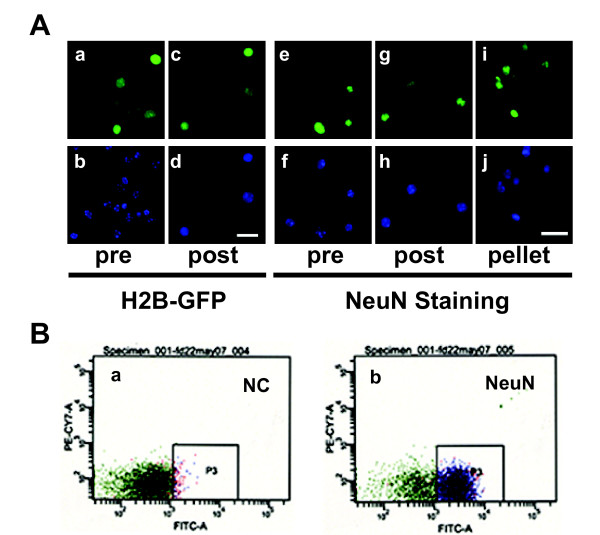
**Neuronal nuclei isolated from adult brain via FACS**. (A) Digitized images of nuclei extracted from forebrain of adult (a-d) CAMIIK-H2B-GFP transgenic mice, and (e-j) wild type mice labeled with NeuN immunoreactivity, as indicated pre and post (FACS sorting), and after pelleting. Green channel for (a, c) GFP or (e, g, i) NeuN; blue channel for DAPI. Notice that post-FACS samples are comprised entirely of neuronal nuclei. Bar = 20 μm. (B) Representative FACS scatter plots from (a) negative control (NC) processed without NeuN antibody, and (b) sample processed with NeuN antibody (NeuN).

**Figure 3:**
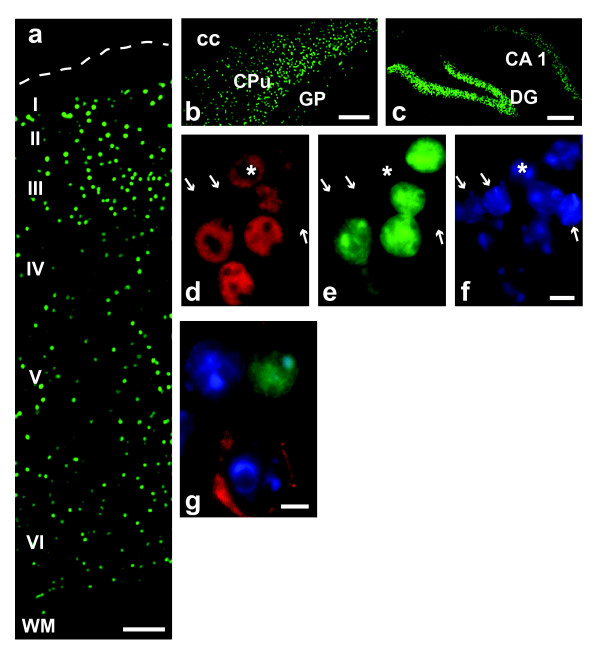
**Neurons expressing H2B-GFP transgene**. Digitized images from 8 week old CAMIIK-H2B-GFP mice. (a) Cerebral cortex. Notice GFP (green) nuclei across layers II-VI, but not in layer I or white matter (WM). (b) caudate-putamen and (c) hippocampus. (d-f) Nuclei extracted from forebrain and processed for NeuN (red), and nucleophilic dye, DAPI (blue). Notice that GFP (green) expression is limited to 5 of the 6 NeuN immunoreactive nuclei. Arrows label GFP-, NeuN- nuclei, * labels GFP-, NeuN+ nucleus. (g) Section from cerebral cortex labeled with anti-GAD67 antibody (red). Notice absence of GFP signal in GABA neuron. Bar is (a, c) 100, (b) 300, (d-g) 5 μm.

### Loss of nuclei during the procedure

Each of the major steps in this protocol – (1) extraction of nuclei from the tissue, (2) ultracentrifugation, (3) immunolabeling and (4) fluorescence-activated sorting – could result in loss of nuclei, together totaling perhaps up to 50% of the neuronal nuclei in the starting material. However, despite of these limitations, the alternative approach, such as nuclei harvesting from primary neuronal culture, is likely not to improve results in a higher yield and would have the additional disadvantage of any *ex vivo *preparation.

### Nucleosomes are preserved in sorted nuclei

**Figure 4:**
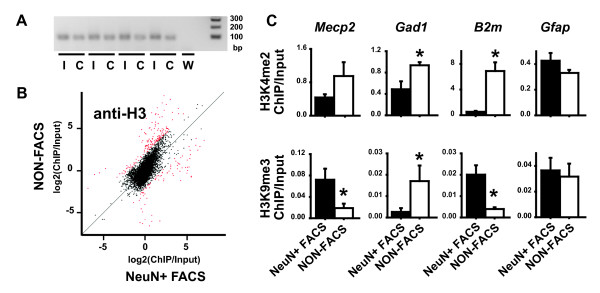
**Neuron-specific chromatin signatures in mouse brain**. (A) Representative gel picture for 25 cycle PCR product (ChIP-GLAS) from 4 independent replicates of Input I and ChIP C, and water control W. (B) Scatter plot showing correlation between Chip-to-input ratios signal from NeuN+ sorted nuclei (NeuN+ FACS) (x-axis) and unsorted nuclei (NON-FACS) (y-axis), using modification-independent antibody against C-terminus of histone H3 anti-H3. (C) Bar graphs showing ChIP-to-input ratio (y-axis) for (top row) dimethylated H3-lysine 4 (H3K4me2) and (bottom row) trimethylated H3-lysine 9 (H3K9me3) as determined for *Mecp2*, *Gad1*, *B2m*, and *Gfap *gene promoters in chromatin of NeuN+ FACS and NON-FACS nuclei from mouse forebrain. Data shown as mean ± S.E.M., N = 6/sorting; *p < 0.05, ANOVA NeuN+ FACS v.s. NON-FACS.

Nucleosomes as the elementary unit of chromatin – are comprised of 146 bp of genomic DNA wrapped around core histones H2A, H2B, H3 and H4. To find out whether nucleosomal structures remain intact in sorted NeuN+ nuclei, we compared nucleosome occupancy at promoters in forebrain from unsorted and NeuN+ nuclei, using a modification-independent anti-H3 C-terminus antibody for ChIP-DSL in conjunction with M8K promoter array (Fig 4A, B). No significant differences between chip-to-input ratios of sorted NeuN+ and unsorted nuclei were observed. Instead, H3 occupancy at specific promoter sequences showed a strong correlation between the NeuN+ and unsorted samples (Spearman rank, r = 0.651370, p < 2.2e^-16^) (Fig. 4B). We conclude that promoter-bound nucleosomes are intact after the sorting procedure.

### Differential histone H3 methylation in neuronal nuclei

To find out whether or not neuronal chromatin, under baseline conditions, shows a histone methylation signature that is different compared to unsorted nuclei, we conducted ChIP for two histone lysine methylation marks, H3K4me2 and H3K9me3. All studies were conducted on the forebrain of male mice 8–12 weeks of age. Histone methylation levels were at 3 out of 4 – arbitrarily chosen – gene promoters significantly different in neuronal chromatin (Fig. 4C). Furthermore, in 2 out of 4 promoters (*B2m, Mecp2*), changes in the "open" mark, H3K4me2 and the "repressive" mark, H3K9me3, were in the opposite direction (Fig. 4C). Notably, H3K9me3 levels at the *Mecp2 *promoter – i.e., the Rett syndrome gene which is robustly and ubiquitously expressed in mature CNS neurons [11-15] – were higher in neuronal vs. unsorted chromatin. This particular finding was unexpected and requires further investigations.

**Figure 5:**
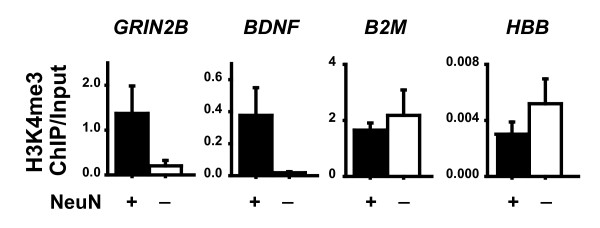
**Neuron-specific chromatin signatures in human prefrontal cortex**. Bar graphs showing ChIP-to-input ratio (y-axis) for trimethylated H3-lysine 4 (H3K4me3) at *GRIN2B*, *BDNF *and *B2M *promoters, and *HBB *(β-globin) locus control region in chromatin of NeuN positive (NeuN+) and negative (NeuN-) FACS sorted nuclei from human prefrontal cortex. Data shown as mean ± S.E.M., N = 3 specimens/sorting. The NeuN+ fraction showed, in comparison to NeuN- fraction of the same subject, increased levels of H3K4me3 at *GRIN2B *and *BDNF *in 3/3 cases. Notice further the extremely low ChIP-to-input ratio for *HBB*.

Next, we wanted to (i) find out whether the above findings can be extrapolated to species other than mouse and (ii) further confirm that histone lysine methylation at select gene loci is differentially regulated in neurons as compared to non-neuronal cells residing in the same tissue. To this end, we utilized postmortem tissue from the human prefrontal cortex for ChIP with H3-tri-methyl-lysine 4 (H3K4me3), a chromatin mark enriched at sites of active gene expression [16,17]. For each tissue sample, NeuN immunopositive and immunonegative nuclei were sorted separately by FACS, and then processed in parallel for anti-H3K4me3 ChIP followed by qPCR for the following genes: (1) The subunit 2B of the NMDA receptor (*GRIN2B*) and (2) brain-derived neurotrophic factor (*BDNF*); both these genes are in postnatal and adult brain predominantly or exclusively expressed in neurons [18,19]. (3) β 2 microglobulin (*B2M*), which in CNS is expressed in a mixed population of neuronal and non-neuronal cells and considered as a "housekeeping gene" in postmortem studies [20,21], and (4) the locus control region of the β globin locus (*HBB*), which is highly regulated in erythopoetic tissues but silent and inactive in brain [22,23]. Levels of H3K4me3 at promoters of the neuron-specific genes, *GRIN2B *and *BDNF*, were consistently higher in chromatin of the NeuN+ nuclei as compared to NeuN- (Fig. 5). These changes were highly specific because H3K4me3 levels in chromatin surrounding *B2M *were higher in NeuN- nuclei, and furthermore, H3K4me3 at the *HBB *locus control region were overall very low (Fig. 5). Taken together, our findings in mouse forebrain and human prefrontal cortex suggest that – even at baseline – important differences exist between histone methylation signatures of neuronal and non-neuronal chromatin.

## Conclusion

The protocol presented here should be particularly useful to investigators who wish to study chromatin regulation, including epigenetic mechanisms of neuronal gene expression, in the mature and the aging brain. The alternative approach, i.e. the study of primary neuronal cultures or neuron-like cell lines, certainly is suited to model developmental mechanisms and neurodevelopmental disease [24-29]. However, neurons and other CNS cells are sustainable in culture, at best, for a few weeks [30,31] and therefore are not ideal from the viewpoint of chronic neuropsychiatric diseases, which often involve a protracted course, late onset and predilection for a specific neuronal subtype. Furthermore, the complexities of brain connectivity and circuitry, among a myriad of other factors, is difficult or impossible to model *ex vivo*. For example, *in vivo *treatment with antipsychotic drugs induces chromatin remodeling in cerebral cortex and striatum, an effect that cannot be mimicked in cortical or striatal cell cultures lacking input from monoamine pathways [32-34]. Finally, chromatin assays in whole brain tissue are potentially confounded by cellular heterogeneity, including potential shifts in cellular composition during the course of aging or due to disease. Given that chromatin immunoprecipitation with genome-wide coverage from as little as 10,000 cells is now feasible [35], it should be possible to use transgenic mice – including the CAMIIK-H2B-GFP lines presented here – to selectively study the epigenome for highly defined subpopulations of neurons in the mouse. Furthermore, the relative resilience of nuclei in frozen-then-thawed postmortem brain tissue makes it possible to apply the techniques presented here to species other than small laboratory animals, including normal and diseased human brain. It remains to be determined whether or not the approach presented here is applicable to tissues other than brain. When compared to sorting protocols that are based on trypsinization of tissue and sorting of intact (neuronal or non-neuronal) somata [36], our approach has several advantages. First, it is applicable to frozen tissues. Second, the yield of recovered nuclei appears to be much higher, albeit direct comparisons are lacking. Third, it is comparatively simple as compared to related protocols.

To summarize, the perspective to develop chromatin assays for specific types of neuronal populations of the mature brain, as described here, will be relevant for a wide range of physiological and neurological conditions related to transcriptional regulation and chromatin remodeling.

## Methods

### An overview of the procedure is provided in Fig. 1

#### Human

Procedures involving human postmortem brain were approved by the institutional review boards of the University of Massachusetts Medical School. Frozen tissues (prefrontal cortex) from 3 specimens (age range: 8–69 years, autolysis interval 5–28 hours) were obtained from the Brain and Tissue Banks for Developmental Disorders, University of Maryland (National Institute of Child Health and Human Development Contract NO1-HD-8-3284) and from a brain bank at the University of California at Davis (Center for Neuroscience, Director Dr. Edward G. Jones).

#### Mice

A cassette, comprised of 8 kB of the *CamIIK *(α subunit) promoter and 1.4 kB of histone H2b-eGFP cDNA (CaMK II-H2B-EGFP), was used to generate 3 lines of transgenic mice by pronuclear injection. All the experiments were performed in strict accordance with Institutional Guidelines regarding use of experimental animals.

### Reagents and reagent setup

Chemicals used include the following: 1 × PBS (Cellgro, 21-040-CV), Triton X-100 (Fluka, 93426), Igepal CA-630 (NP-40) (Sigma, 1–3021), Protease inhibitor cocktail tablets (Roche, 11697498001), Phenylmethanesulfonyl Fluoride (PMSF) (Sigma, P7626), Mouse anti-NeuN (Upstate, MAB377), Goat anti-mouse IgG, Alexa Fluor 488 (Invitrogen, A11029), Bovine serum Albumin (BSA) (Sigma, B-4287), Normal goat serum (Vector, S-1000), Micrococcal nuclease (Sigma, N-3765), Protein G Agarose, (Upstate, 16–266), Rabbit Anti-H3 (Abcam, ab1791), Rabbit Anti-H3K4me2 (Upstate, 07–030), Rabbit Anti-H3K9me3 (Upstate, 07–442), Rabbit Anti-H3K4me3 (Upstate, 07–473), Normal rabbit IgG (Upstate, 120370)

Nuclei Extraction Buffer (NEB): 0.32 M Sucrose, 5 mM CaCl_2_, 3 mM Mg(Ac)_2_, 0.1 mM EDTA, 10 mM Tris-HCl (pH8), 1 X Protease inhibitor cocktail, 0.1 mM PMSF, w/o 0.1% Triton X-100, w/o 0.1% NP-40. Sucrose Cushion: 1.8 M Sucrose, 3 mM Mg(Ac)2, 10 mM Tris-HCl (pH8). Blocking Solution: 0.5%. Bovine serum albumin (BSA), 10% normal goat serum, in 1 × PBS.

### Nuclei extraction from brain tissue

Total nuclei were extracted via sucrose gradient ultracentrifugation. In the work presented here (mouse), each sample was derived from a single forebrain (adult males, 8–12 weeks of age); (human) 1000 mg of cerebral cortex. All the reagents used were pre-chilled and the entire procedure was performed on ice. Fresh or frozen samples were homogenized by douncing 50 times in 5 mL NEB with 0.1% Triton X-100, or alternatively, 0.1% NP-40. Triton X-100 was preferable if nuclei require immunotagging (with NeuN, for example), while NP-40 as a milder detergent left more nuclei intact and sufficient when working with nuclei expressing GFP. After douncing, brain homogenates were transferred into 14 mL ultracentrifuge tubes (Beckman, 14 × 95 mm, 344061), and 9 mL of Sucrose Cushion was carefully loaded directly to the bottom of the ultracentrifuge tube. Ultracentrifugation was performed at 24400 rpm for 2.5 hrs at 4°C (Beckman, L8-70M, SW28 rotor). After centrifugation, a nuclei pellet – thin and typically with a light yellow taint – was formed on the bottom of the tube. The sticky white tissue debris was restrained in the middle interface of the two sucrose layers. Supernatant, including debris, was carefully removed. 1200 μl of chilled 1 × PBS was then added into the tube and incubated without disturbing the pellet, for 20 min on ice. This incubation is recommended because the nuclei pellet is easier to dissociate and consequently, there will be less damage to the nuclei. After incubation, the nuclei were dissociated by pipetting up and down. FACS required single nuclei in solution but excess pipetting tended to break nuclei, at least in the unfixed preparations. Pipetting required optimization by checking the nuclei "quality" (intact single nuclei, non-adhering, without debris) under the light microscope. The total number of nuclei was counted by using a hemacytometer. One mouse forebrain typically yielded 20–30 × 10^6 ^unfixed nuclei after ultracentrifugation.

One alternative option was to fix the nuclei by directly douncing the tissue in NEB containing 1% formalin. Typically, we fixed with formalin at room temperature for 5–10 min, and then added glycine to a final concentration of 125 mM. The fixed nuclei were pelleted by centrifugation, re-suspended in 5 mL of NEB, and subjected to ultracentrifugation as described above. Nuclei clumping during isolation was – in our experience – usually to blame if the final yield was lower than expected. For unfixed samples, factors that appear to promote clumping are (i) contamination from brain debris, or (ii) DNA leaking from broken nuclei, which appears to be alleviated via careful handling and performing the whole procedure on ice. When a fixation step was included (see above), the only way to significantly alleviate the clumping was to remove Ca^2+ ^from the NEB, albeit this increased the proportion of broken and damaged nuclei. Thus, lower post-FACS yield is to be expected when working with fixed samples.

### Immunotagging of neuronal nuclei

Next, neuronal nuclei were tagged by immnofluorescence staining, using an anti-NeuN antibody (Fig. 2A, panels e-j). Firstly, anti-NeuN (Ms) and anti-Ms IgG (Alexa 488) antibodies were co-incubated at room temperature for 5 min (Table 1), then nuclei solution was added and the mixture incubated in darkness for at least 20 min at 4°C before FACS. The proportion of NeuN positive nuclei in the material recovered after ultracentrifugation was about 50%. The FACS background signal was minimal in these preparations (Fig. 2B, panel a).

**Table 1:** Antibodies used for immunotagging of neuronal nuclei.

	Primary/secondary antibody conjugate				
		
	anti-NeuN (Ms)	anti-Ms IgG Alexa 488	Blocking Solution	1 × PBS	Nuclei solution
NeuN	1.2 μl	1 μl	100 μl	300 μl	1000 μl
Negative control	0 μl	0.2 μl	20 μl	60 μl	200 μl

### Neuronal nuclei isolation via FACS

Next, the fluorescent nuclei (H2B-GFP transgene or NeuN labeled) were filtered through a 40 μm Nitex mesh to remove any remaining clumps, and then run through a FACS machine (Vantage SE/Diva, BD Biosciences) with proper gates settings based on the size and density of nuclei, to ensure that only single NeuN+ nuclei were sorted, which typically included 40% of the whole population (Fig. 2B, panel b). After FACS, the NeuN+ nuclei were reanalyzed to confirm the purity. Sample purity typically is > 95%. For selected samples, the purity of NeuN+ nuclei was checked under the microscope (Fig. 2A, panels c, d and g, h). FACS sorting should be performed on the same day as extraction and NeuN labeling of nuclei. For a subset of experiments, including those involving human postmortem brain, immunonegative (NeuN-) nuclei were collected and processed in parallel to the fraction of neuronal (NeuN+) nuclei.

### Expected yield of neuronal nuclei

We usually obtained – from a single mouse forebrain – 2.5 × 10^6 ^NeuN+ nuclei post-FACS if fixative was added to the NEB and up to 8 × 10^6 ^nuclei if processed unfixed. We usually obtained from 1000 mg of human (prefrontal) cerebral cortex 5 × 10^6 ^NeuN+ nuclei if processed unfixed.

### Post-FACS nuclei pelleting

After FACS, nuclei were collected in a large volume of 1 × PBS, roughly 4 × 10^6 ^nuclei in 10 mL of 1 × PBS. This was not ideal for ChIP because processing such a large volume is associated with very high expense and potential "wasting" of antibodies and other reagents. Therefore, pelleting was important, but the challenge thereby was that post-FACS nuclei became fragile and most of them were destroyed by centrifugation in 1 × PBS. Therefore, we tested different buffers and noticed that the presence of Ca^2+ ^and Mg^2+ ^is crucial for integrity of nuclei during pelleting. Hence, the concentration of sucrose, Ca^2+^, and Mg^2+ ^in post-FACS nuclei solution was adjusted by adding the following to each 10 mL of post-FACS nuclei: 2 mL of 1.8 M Sucrose, 50 μl of 1 M CaCl_2_, and 30 μl of 1 M Mg(Ac)_2_. Samples were mixed gently, incubated on ice for 15 min, and centrifuged at 3000 rpm for 15 min at 4°C.

### Chromatin immunoprecipitation

The FACS sorted nuclei were prepared either by micrococcal nuclease digestion for native chromatin immunoprecipitation (NChIP) as described [37], or in case of fixed nuclei, sheared by sonication (Branson Sonifier 250) for XChIP. (Typically, samples were sonicated at power level 6 (Branson, Danbury CT) in ice water, by applying 10 runs of 30 sec impulse with 1 min resting interval. Again, sonication conditions need to be optimized before each experiment.) In the work presented here, only NChIP was used. In brief, micrococcal nuclease (MNase) digestion was performed with a working concentration of 4U/mL at 37°C for 5 min. The resulting mono-nucleosomal preparation (around 146 bp) [see Additional file 1, panel A] was precleaned by incubating with protein G agarose (which should also remove the antibodies used for NeuN labeling of nuclei) and then subjected to immunoprecipitation using antihistone antibodies (see Reagents). Control experiments included samples processed with normal rabbit IgG, in parallel to samples with specific antibodies. Typically, we used 0.5 – 8 × 10^6 ^nuclei as input.

### Real time PCR

Quantification of DNA extracted from immunoprecipitates was done by real time PCR using custom-designed primers targeting the promoter regions of the following genes: (mouse) methyl CpG binding protein 2 (*Mecp2*), glutamate decarboxylase 1 (*Gad 1*), β2-microglobulin (*B2m*), and Glial fibrillary acidic protein (*Gfap*); (human) glutamate receptor, ionotropic, N-methyl D-aspartate 2B (*GRIN2B*), brain derived neurotrophic factor (*BDNF*), β2-microglobulin (*B2M*), and the locus control region of the globin genes (*HBB*). Mouse*Mecp2 *primer sequence (119 bp): forward GCCTCTTTTCCCTGCCTAAA, reverse CCCTTGCTCTTTGTCGAGAT; *Gad 1 *(98 bp): forward TGTCTCACCAAAGTCCCTGTC, reverse CACGTCTGGTTCGGTGTCT; *B2m *(112 bp): forward GGGAAAGTCCCTTTGTAACCT, reverse GCGCGCGCTCTTATATAGTT; *Gfap *(100 bp): forward TACCAGAAAGGGGGTTCCTT, reverse AACTCCTCTCACCCCACTGA. Human*GRIN2B *primer sequence (64): forward TCCTCTTCCATTCAGGTTGG, reverse GGCTATACCATTCCTGGGACA; *BDNF *(102): forward AGCCCAACAACTTTCCCTTT, reverse GAGAGCTCGGCTTACACAGG; *B2M *(99): forward GGGCACCATTAGCAAGTCAC, reverse GGCGCTCATTCTAGGACTTC; *HBB *(87): forward CCCCAGGTAGTTCCCTTT reverse TTCAAGGCCCTGTAGTTGCT. Quantification was done as described [37]. For each of the antibodies used in this study (anti-H3K4me2, -H3K9me3, and -H3K4me3), specific signal was limited to input and chip fractions, and differed from controls by at least 3–4 cycle thresholds [see Additional file 1, panel B, C].

### ChIP-on-Chip

To study histone occupancy at gene promoters on a larger scale, a mouse promoter array, M8K (Aviva Systems Biology, San Diego, CA), containing 8000 40-mer oligonucleotide probes targeting gene promoters, was used. The DNA samples were amplified and labeled by Aviva's ChIP-GLAS system. Briefly, both input and immunoprecipated (ChIP) DNA were biotinylated and annealed to the M8K-Oligo-Mix. The DNA samples were then amplified by ligation mediated PCR with T3 and T7 based primers (25 cycles), labeled with Atto 550 or 647, and hybridized onto the chip. Amplification, labeling, hybridization, and washing was carried out according to the manufacturer's instruction (Aviva, AK-0524). Slides were scanned with DNA Microarray ScannerBA (Agilent Technologies, Santa Clara, CA, USA) and intensities extracted with Feature Extraction Software Version 9.1 (Agilent Technologies, Santa Clara, CA, USA). Raw data were read into the R statistical computation environment for preprocessing and data analysis (R: Development core team (2004). R: A language end environment for statistical computing. Vienna, Austria.URL found in Availability and requirements section). Local background intensities were subtracted from raw signals and negative values were replaced with small positive ones. Signals were then normalized using a variance stabilization method described in [38,39] and implemented in the vsn Bioconductor package (Gentleman, RC. et. al., Bioconductor: open software development for computational biology and bioinformatics. Genome Biology 5, R80 (2004)).

### Other amplification procedures

We used the Genomeplex whole genomic amplification kit (WGA2, Sigma) to amplify DNA immunoprecipitates, which resulted in 2 to 5 ug of (amplified) DNA from starting material (10 ng of input DNA; ChIP DNA from 0.5 × 10^6 ^nuclei).

## Availability and requirements

R:A language end environment for statistical computing:  

## Authors' contributions

YJ, H-SH and SA contributed to conception and study design, YJ, H-SH and AM conducted experiments and data analyses; JS performed microarray data analyses; YJ and SA wrote the paper.

## Supplementary Material

Additional file 1 (A) Images from ethidium bromide-stained 1.3% agarose gels showing chromatin DNA from mouse forebrain before (MNase-) and after (MNase+) micrococcal nuclease (MNase) digestion. All samples were treated with RNase A. The DNA ladder is shown on the left side of gel picture. Notice approximately 146 bp DNA fragment only in MNase+ samples. (B), SYBR-green based melting curves from immunoprecipitates with anti-H3K4me2 antibody using primer pairs for mouse *B2m *and *Gad1*; notice single peak for specific product. (C) Representative amplification curves of inputs (black circles), immunoprecipitates (red circles) and IgG control (green circles), dotted line indicating cycle threshold. Data shown for *Gad1 *and *B2m *separately. Notice samples processed with non-specific IgG show much higher cycle thresholds than input and immunoprecitats. Click here for file
