# Supplementary material for: Isolation of neuronal chromatin from brain tissue
Source: BMC Neurosci. 2008 Apr 28;9:42. doi: 10.1186/1471-2202-9-42 (PMC2377267; doi:10.1186/1471-2202-9-42)
Supplement: Additional file 1 — (A) Images from ethidium bromide-stained 1.3% agarose gels showing chromatin DNA from mouse forebrain before (MNase-) and after (MNase+) micrococcal nuclease (MNase) digestion. All samples were treated with RNase A. The DNA ladder is shown on the left side of gel picture. Notice approximately 146 bp DNA fragment only in MNase+ samples. (B), SYBR-green based melting curves from immunoprecipitates with anti-H3K4me2 antibody using primer pairs for mouse B2m and Gad1; notice single peak for specific product. (C) Representative amplification curves of inputs (black circles), immunoprecipitates (red circles) and IgG control (green circles), dotted line indicating cycle threshold. Data shown for Gad1 and B2m separately. Notice samples processed with non-specific IgG show much higher cycle thresholds than input and immunoprecitats. [file 1471-2202-9-42-S1.pdf]

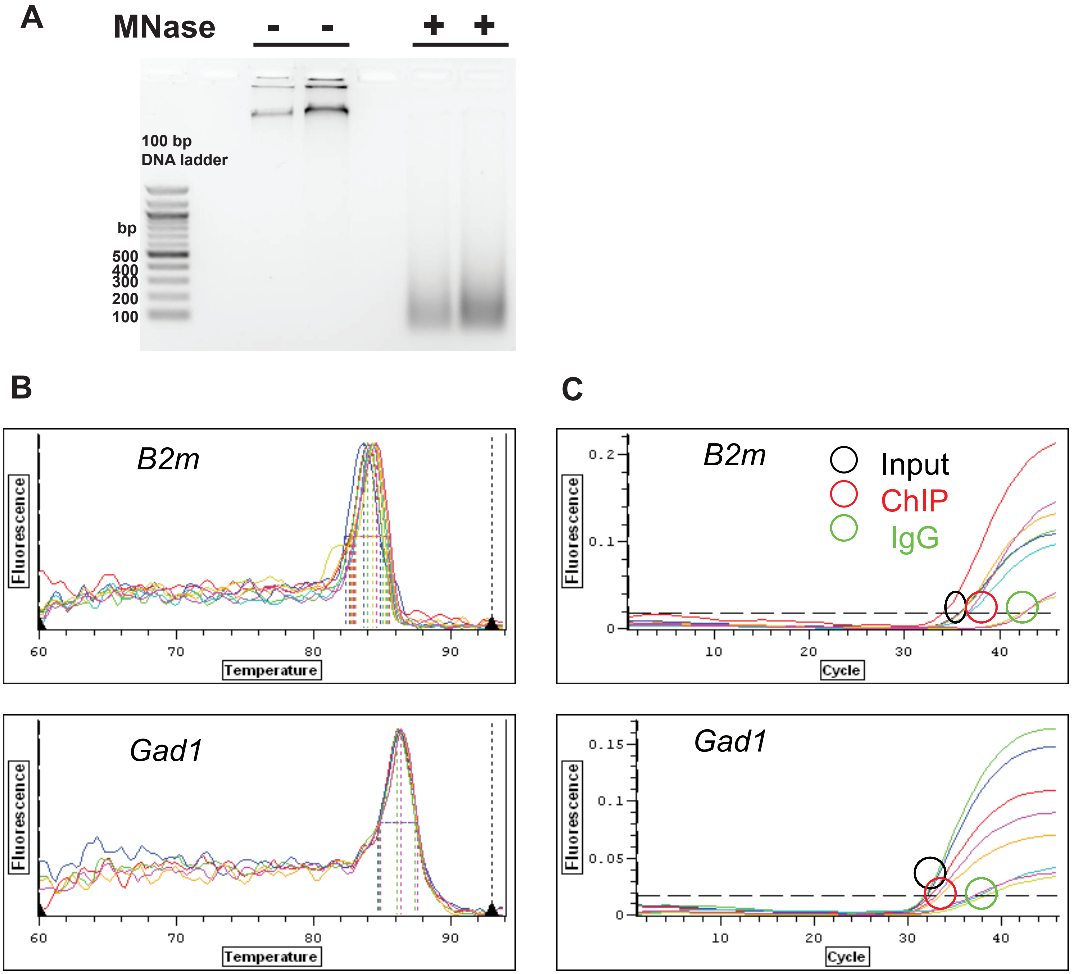

Supplementary Fig. 1:
(A) Images from ethidium bromide-stained 1.3 % agarose gels showing chromatin DNA from mouse forebrain before (MNase-) and after (MNase+) micrococcal nuclease (MNase) digestion. All samples were treated with RNase A. The DNA ladder is shown on the left side of gel picture. Notice approximately 146 bp DNA fragment only in MNase+ samples.
(B), SYBR-green based melting curves from immunoprecipitates with anti-H3K4me2 antibody using primer pairs for mouse *B2m* and *Gad1*; notice single peak for specific product.
(C) Representative amplification curves of inputs (black circles), immunoprecipitates (red circles) and IgG control (green circles), dotted line indicating cycle threshold. Data shown for *Gad1* and *B2m* separately. Notice samples processed with non-specific IgG show much higher cycle thresholds than input and immunoprecitates.
